# Asymptomatic Primary Tumor Resection in Metastatic Colorectal Cancer: A Systematic Review and Meta-Analysis

**DOI:** 10.3389/fonc.2022.836404

**Published:** 2022-03-29

**Authors:** Yefei Shu, Ling Xu, Wei Yang, Xiaofeng Xu, Song Zheng

**Affiliations:** ^1^ Department of Medical Oncology, Hangzhou Cancer Hospital, Hangzhou, China; ^2^ Department of Oncology and Hematology, Hangzhou Red Cross Hospital, Hangzhou, China; ^3^ Department of Medical Oncology, Key Laboratory of Clinical Cancer Pharmacology and Toxicology Research of Zhejiang, Affiliated Hangzhou First People’s Hospital, Zhejiang University School of Medicine, Hangzhou, China

**Keywords:** colorectal cancer, asymptomatic, primary tumor, resection, meta-analysis

## Abstract

**Background:**

In patients with metastatic colorectal cancer (mCRC) with an asymptomatic primary tumor, there is no consensus on the indication for resection of the primary tumor.

**Methods:**

The PubMed, Embase and the Cochrane Library databases were searched from inception to November 30,2021. A meta-analysis was performed using RevMan (version 5.3.3; The Cochrane Collaboration) on the outcome of mCRC patients with or without resection of the primary tumor in 8 selected studies.

**Results:**

This meta-analysis included 2805 colorectal cancer patients with an asymptomatic primary tumor from 8 selected studies. Primary tumor resection (PTR) patients had longer overall survival (OS: MD =6.76 [3.39, 10.12], I^2^ = 77%, P < 0.0001), compared with non-primary tumor resection (NPTR) patients. In the subgroup, the randomized controlled trials (RCT) PTR group didn’t have longer overall survival (OS: MD =3.79 [-3.49, 11.08], I^2^ = 69%, P= 0.31); the Non-RCT PTR group had longer overall survival (OS: MD =8.42 [3.14, 13.70], I^2^ = 89%, P= 0.002). In the meanwhile, compared with NPTR group, the 2-year overall survival rate, the 3-year overall survival rate, 5-year overall survival rate in the PTR group is higher (OR=2.35 [1.74, 3.18], I^2^ = 0%, P < 0.00001; OR=3.61 [2.35, 5.54], I^2^ = 0%, P < 0.00001; OR=3.02 [1.72, 5.33], I^2^ = 48%, P= 0.0001, respectively).

**Conclusions:**

Our results from studies demonstrate that the resection of primary tumor is a prognostic factor for survival in mCRC patients. However, 2 RCTs showed the resection of primary tumor was not related with a significant survival benefit in subgroup. Therefore, a larger RCT in the era of modern chemotherapy and liver resection techniques would be helpful.

## Introduction

Clinically, synchronous liver metastasis of colorectal cancer can be divided into four types according to whether the primary tumor has clinical symptoms and whether the liver metastasis can be resected: (1) the primary tumor has no symptoms and the liver metastasis can be resected, (2) The primary lesion is asymptomatic and liver metastases are unresectable, (3) The primary lesion was symptomatic and the liver metastases can be resected, (4) The primary lesion is symptomatic and the liver metastases are unresectable. There is no dispute about the treatment principle of the latter two situations. The primary lesion should be treated first (or liver metastasis should be treated at the same time). In the first situation, the treatment involving preoperative chemotherapy, simultaneous or staged resection will be not discussed here. For the second situation, should we first convert to chemotherapy, or should we first remove the primary tumor?

At present, most studies suggest that primary resection is better than chemotherapy alone in asymptomatic colorectal cancer patients with liver metastasis. In a population-based cohort research, palliative primary tumor resection was related to improved overall and cancer-specific survival (1). The benefit that resection of the primary tumor in asymptomatic patients with metastatic colorectal cancer (mCRC) improved survival independent of other prognostic variables was more pronounced in stage IVA patient (2). Another study also indicated that the resection of primary tumor was a prognostic factor for survival in stage IV CRC patients (3). Compared to patients who received chemotherapy as the first treatment, mCRC patients with synchronous unresectable metastases who underwent primary tumor resection (PTR) followed by chemotherapy had significantly longer survival times (4).

However, the efficacy of the PTR for unresectable mCRC has been controversial (5). A study demonstrated that compared to patients receiving chemotherapy alone the overall risk of death was significantly higher after elective surgery in palliative treatment of asymptomatic mCRC. In other words, there is no substantial difference between these treatments (6).Moreover, PTR in patients with asymptomatic primary tumor and unresectable metastases was not associated with an improvement in overall survival (OS) after propensity score matching (7). PTR is not recommended for the reason that PTR was not associated with improved survival compared with systemic chemotherapy among patients with unresectable metastatic colon cancer (8). In a conclusion, PTR should no longer be considered a standard therapy for patients with CRC with asymptomatic primary tumors and synchronous unresectable metastases (9).

Patients with asymptomatic mCRC represent a significant heterogeneous group. The aim of our study was to analyze the studies, which compared the clinical outcomes of patients with asymptomatic mCRC and un-resectable metastases who had PTR with those without resection. There is no consensus about the solution. In this paper, meta-analysis was performed on the retrospective study and randomized controlled study on the benefit of PTR.

### Search Strategy

**Figure 1:**
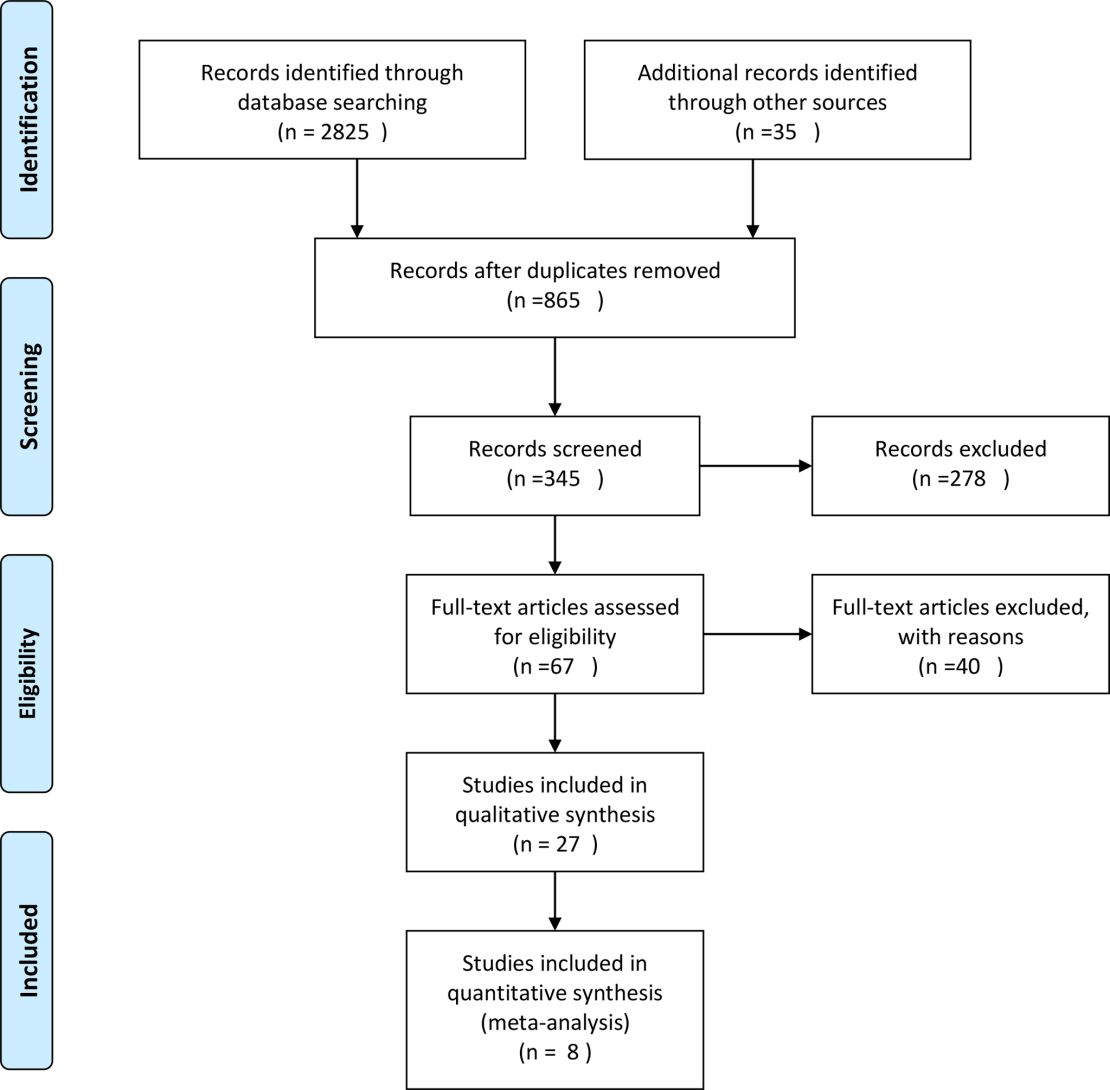
PRISMA Flow Diagram.

The PubMed, Embase and the Cochrane Library databases were searched from inception to November 30,2021 for relevant studies using specified eligibility criteria. Only human-related studies published from 2013 to 2021were included. The search terms included two combinations: combination 1 (Primary Asymptomatic Tumor Resection) AND (colorectal cancer); and combination 2 (colorectal cancer) AND (unresectable OR stage IV) AND (Resection); with the search filter: clinical trial, abstract, title-abstract. Titles and abstracts were screened for all studies, and full text was obtained for those meeting the inclusion criteria. Disagreements were resolved by consensus. PRISMA Flow Diagram is shown in **Figure 1**.

### Inclusion Criteria

Inclusion criteria were: enrolled adults (age ≥18); comparative study, patients with mCRC, primary PTR as one intervention and chemotherapy alone as the other. Only article in English were selected. Finally, 8 studies were determined eligible for meta-analysis.

### Exclusion Criteria

Researches are excluded if they were reviews, letters, case reports, case series, comments, editorials, or observational studies, as well as non-English studies. Studies including less than 40 patients were excluded.

### Outcomes

The primary outcome measures for the studies included was OS. Secondary outcomes were 2-year OS rate, 3year OS rate, and 5-year OS rate. Two authors determined the eligibility of all retrieved studies independently, and discrepancies were resolved through consultation with a third reviewer.

### Assessment of Risk of Bias and Quality of Evidence

**Figure 2:**
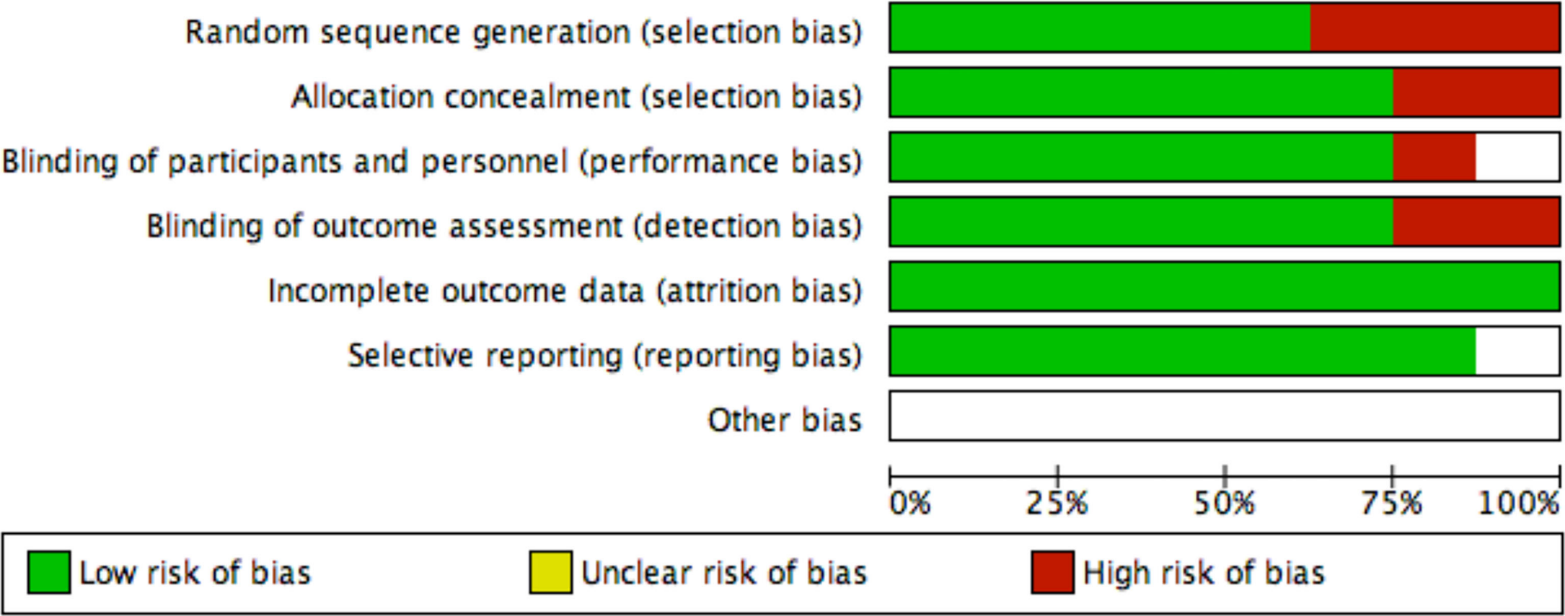
Risk of bias graph.

Yefei Shu and Ling Xu independently assessed the quality of all included trials by using the Cochrane Collaboration risk of bias tool. We also examined the quality of evidence for outcomes using the grading of recommendations assessment and evaluation (GRADE) approach (**Figure 2**).

### Data Synthesis

Statistical analyses were performed using RevMan (version 5.3.3; The Cochrane Collaboration). Analyses for all outcomes were conducted on an intention to treat basis. We used odds ratios and their associated 95% confidence intervals to assess outcomes, and considered a P value less than 0.05 to be statistically significant. We assessed heterogeneity using the I^2^ test. If significant heterogeneity was not present (I^2^<50%), we used fixed effects models to pool outcomes; we used random effects models when significant heterogeneity was present (I^2^≥50%). The possibility of small study effects was assessed qualitatively by visual estimate of the funnel plot and quantitatively by calculation of the Egger test.

### Subgroup Analyses

A subgroup analysis was performed to test interactions according to study design (RCT and Non-RCT).

### Sensitivity Analyses

Sensitivity analyses were conducted by excluding trials with high or unknown risk of bias; excluding trials with high risk or unknown risk of bias of the different domains; excluding trials with a follow-up of less than one year; using random effect models.

## Results

**Figure 3:**
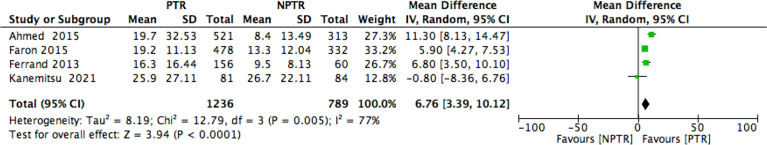
OS Forest plot.

**Figure 4:**
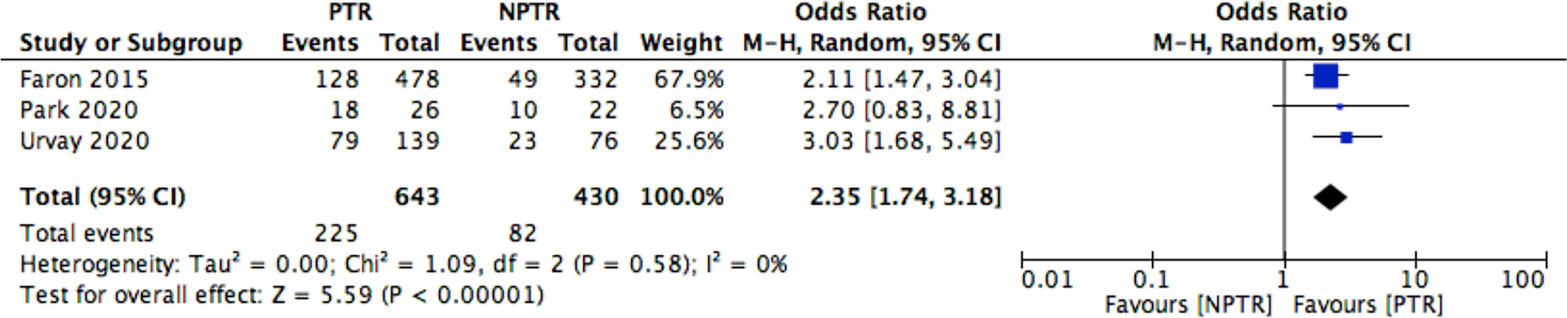
2-year OS rate Forest.

**Figure 5:**
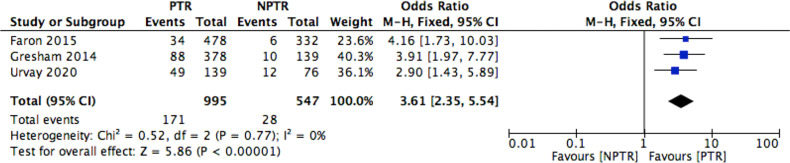
3-year OS rate Forest.

**Figure 6:**
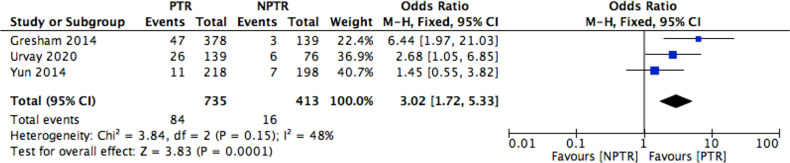
5-year OS rate Forest.

This meta-analysis included 2805 colorectal cancer patients with an asymptomatic primary tumor from 8 studies. Characteristics of included studies and outcome events are shown in **Table 1**. Compared with non-primary tumor resection (NPTR) patients, PTR patients had longer overall survival (2, 9–11) (OS: MD =6.76 [3.39, 10.12], I^2^ = 77%, P < 0.0001) (**Figure 3**). In the meanwhile, compared with NPTR group, the 2-year overall survival rate (10, 12, 13), the 3-year overall survival rate (10, 13, 14), 5-year overall survival rate (7, 13, 14)in the PTR group is higher (OR=2.35 [1.74, 3.18], I^2^ = 0%, P < 0.00001; OR=3.61 [2.35, 5.54], I2 = 0%, P < 0.00001; OR=3.02 [1.72, 5.33], I2 = 48%, P= 0.0001, respectively) (**Figures 4**–**6**).

**Table 1:** Characteristics of included studies and outcome events.

Study name	Study design	Country	Mean age (years)	No (PTR Vs NPTR)	Sex (M/F)	Research time (years)	Median Follow-up (months)	Outcome
Ahmed 2015 (2)	cohort	Canada	PTR: 69 NPTR: 71	834(521/313)	PTR:297/224 NPTR: 186/127	1992-2005	150	OS
Yun 2014 (7)	Retrospective	Korea	PTR: 58 NPTR: 59	416(218/198)	PTR:141/77 NPTR: 130/68	2000-2008	_	5-year OS rate
Kanemitsu 2021 (9)	RCT	Japan	65	165(81/84)	PTR:45/36 NPTR: 45/39	2012-2019	22.0	OS
Faron 2015 (10)	Retrospective	France	PTR: 64 NPTR :62	810(478/332)	PTR: 293/185 NPTR: 211/121	FFCD-9601: 1997–2001, FFCD-2000-05: 2002–2006, ML-16987: 2003–2004, ACCORD-13: 2006–2008	33	OS, 2-year, 3-year OS rates
Ferrand 2013 (11)	RCT	France	PTR: 64 NPTR: 62	216(156/60)	PTR:96/60 NPTR: 44/16	1997-2001	33	OS
Park 2020 (12)	RCT	Korea	PTR: 62.3 NPTR: 58.8	48(26/22)	PTR:21/5 NPTR: 12/10	2013-2016	15.0	2-year OS rate
Urvay 2020 (13)	Retrospective	Turkey	PTR: 59 NPTR: 62	215(139/76)	PTR:85/54 NPTR: 51/25	2009-2016	until 1 March 2019	2-year, 3-year and 5-year OS rates
Gresham 2014 (14)	Cohort	Canada	PTR: 66.5 NPTR: 70	517(378/139)	PTR:195/183 NPTR: 87/52	2006-2008	80	3-year, 5-year OS rates

OS, Overall survival.

**Figure 7:**
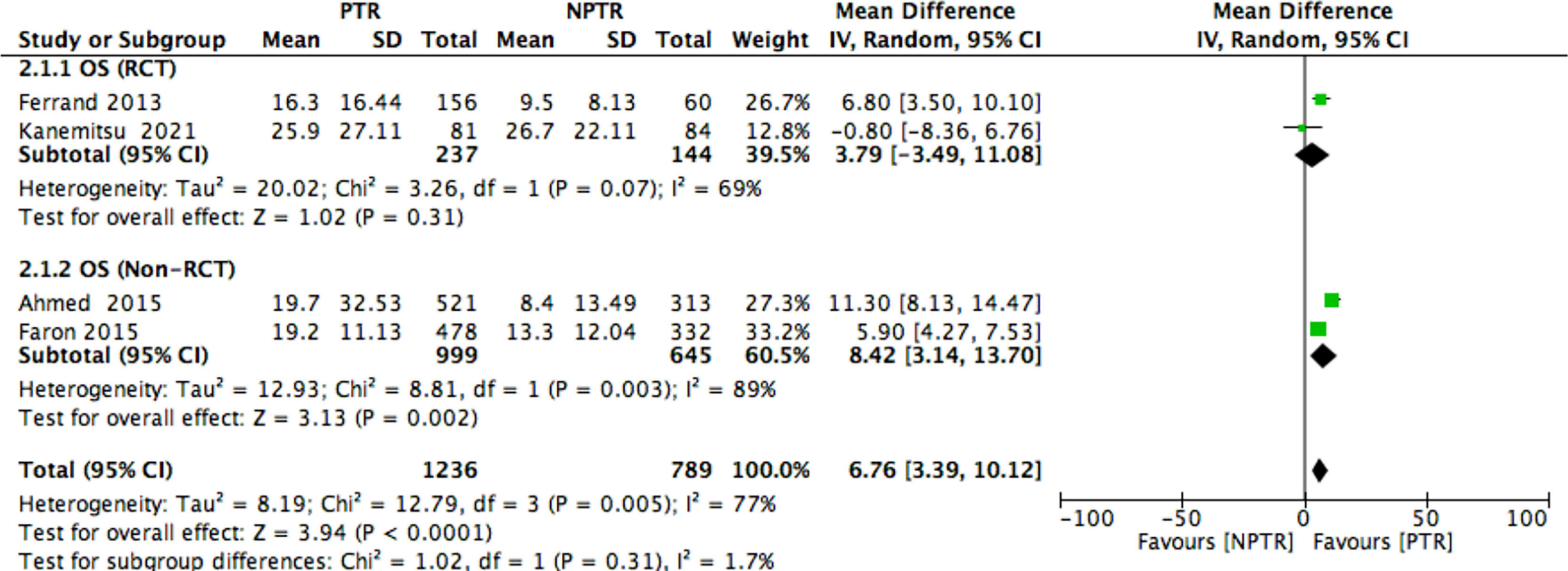
Subgroup analysis of OS Forest plot.

Subgroup analyses found that the randomized controlled trials (RCT) PTR group didn’t have longer overall survival (9, 11) (OS: MD =3.79 [-3.49, 11.08], I^2^ = 69%, P= 0.31); the Non-RCT PTR group had longer overall survival (2, 10) (OS: MD =8.42 [3.14, 13.70], I^2^ = 89%, P= 0.002) (**Figure 7**).

## Discussion

There is no consensus on the indication for PTR in patients with mCRC with asymptomatic primary tumor. However, PTR remains common practice for patients with synchronous mCRC. In addition, treatment disparities are also associated with socioeconomic as well as clinicopathologic factors (15). PTR in conjunction with postoperative chemotherapy among stage IV colon cancer patients with unresectable metastases was associated with a longer survival benefit compared with other therapy strategies (16). In the majority of the previous reports, PTR was correlated to improved survival and to the possibility for a better response to postoperative chemotherapy (17). Both PTR and up front chemotherapy appear appropriate initial management strategies. However, a trend towards an overall survival advantage with PTR, for the reason that the procedure has a low post-operative mortality, and that most complications are transient and minor (18). PTR in patients with synchronous metastasized CRC is controversial, although data suggested that resection might be a positive prognostic factor for survival (19). In multivariate survival analysis, PTR was associated with a significant survival benefit (HR 0.59, p < 0.001) (20).

However, this conclusion has been widely criticized for the following reasons: (1) the proportion of patients with tumor stage IV B is low, the tumor load is relatively small, and the proportion of colon cancer is higher (2) The patients who received surgery had better general condition, lower performance status score and more chances to receive multi line chemotherapy and targeted therapy (3)Some studies included patients who underwent radical resection of primary lesions and liver metastases (4) all studies included are non RCT studies. Therefore, the evidence level of this conclusion is not high.

Korean scholar Yun JA et al. (7) Retrospectively analyzed 259 cases of asymptomatic unresectable synchronous colorectal cancer with liver metastasis. Multivariate analysis found that tumor located in the rectum and tumor diameter greater than 5cm were independent risk factors for intervention. Nitzkorski Jr (21), an American scholar, summarized the data of 143 patients with asymptomatic primary stage IV colorectal cancer who first received systemic chemotherapy in our center. Multivariate analysis showed that there was no independent risk factor for primary symptoms. Overall, in the era of modern chemotherapy and targeted therapy, the probability of primary tumor symptoms requiring surgical intervention has a downward trend.

In order to reduce the bias of potential case selection, MD Anderson scholar alawadi Z et al. (8). Used experimental variable analysis and landmark method (patients with survival longer than 12 months were included in the statistical analysis). Results a total of 15154 patients were included in the study, of which 8641 patients (57%) underwent primary tumor resection. Cox regression analysis and propensity score analysis showed that the overall survival of primary tumor resection group was significantly prolonged, However, after adjustment by experimental variable analysis and landmark method, the survival advantage of primary tumor resection group disappeared.

Recently, some scholars have proposed that patients with unresectable left colon liver metastasis are prone to acute complications such as obstruction, bleeding and perforation, which need surgical or endoscopic treatment; In addition, the biological behavior of patients with left colon is better than that of patients with right colon, and the proportion of *RAS* mutation is low, so they have more opportunities to receive anti *EGFR* treatment; Therefore, patients with unresectable liver metastasis from left colon cancer may benefit more from primary resection than patients with right colon cancer. Zhang RX (22)of the cancer center of Sun Yat sen University in Guangzhou analyzed the clinicopathological data of 194 patients with asymptomatic primary tumor and unresectable synchronous colorectal cancer with liver metastasis from 2007 to 2013. Among them, 125 patients received palliative primary tumor resection and 69 patients only received systemic chemotherapy, Multivariate analysis showed that the location of primary tumor was the only risk factor for survival (RR 0.569, P = 0.007). Further subgroup analysis showed that for the right colon group, the only independent risk factor affecting the prognosis was histological type; For the left colon group, the independent prognostic factors included histological type, multiple liver metastases and palliative resection. The authors suggest that primary resection of the left colon with unresectable mCRC can significantly prolong survival, but it needs to be further confirmed by prospective RCT studies.

In the course of modern chemotherapy and targeted treatment, the incidence of primary symptoms in asymptomatic colorectal cancer patients with non resectable liver metastasis is lower. Although retrospective studies suggest that palliative resection of primary focus can prolong the survival of patients, especially in the left hemicolon, there is a significant bias in these studies. Therefore, this conclusion needs to be treated carefully and more prospective RCT studies are needed to confirm.

In 2008, Eisenberger, A reviewed the medical literature from 1996–2006 using the search terms metastatic colorectal cancer and primary resection to find non-curative resection of asymptomatic colorectal primary tumors may prolong survival (23). In 2015, Faron, M. et al. made a pooled analysis of individual data from four randomised trials and fonud that primary tumour resection was independently associated to a better OS in patients with CRC and unresectable synchronous metastases (10). Currently, aggressive surgical treatments should be integrated with all the available non-surgical options to maximize disease control and patient survival (24). Furthermore, multidisciplinary team approaches may be helpful in finding the suitable therapy. In case of surgical resection, minimally invasive surgery is recommended (25). In a selected population of patients with colon cancer and unresectable synchronous distant metastases, immediate colectomy followed by chemotherapy in association with targeted therapy was associated with longer OS. This strategy appears to be the most appropriate, especially for those with good performance status, well-differentiated tumors, and synchronous liver metastases only (26). In mCRC patients with unresectable metastases receiving chemotherapy, up-front PTR was independently associated with prolonged OS. Patients eligible for secondary metastases resection and/or bevacizumab may benefit the most from PTR (27). On the contrary, resection of the primary tumour in asymptomatic patients with unresectable mCRC who are managed with chemo/radiotherapy is not associated with a consistent improvement in overall survival. In addition, resection does not significantly reduce the risk of complications from the primary tumour (i.e. obstruction, perforation or bleeding) (28). The rate of complications related to the non-resected colorectal tumor is very low using oxaliplatin as first line chemotherapy. Non-operative management of asymptomatic CRC with un-resectable liver metastases is a safe approach (29). Some people hold the view that asymptomatic patients with mCRC do not routinely need to undergo resection of the primary tumor. Although several retrospective analyses suggest that patients who undergo resection of the primary tumor live longer, most of these reviewed data prior to the advent of modern polychemotherapy and are subject to considerable bias, as patients who were considered able to undergo surgery likely had better overall prognoses than those who were not (30).

Our results from studies indicate that resection of the primary tumor is a prognostic factor for survival in mCRC patients. However, 2 RCTs showed resection of the primary tumor was not associated with a significant survival benefit in subgroup. The resection of the primary tumor was a prognostic factor for longer survival in non-randomized trial but was not associated with better survival in randomized trial. This finding may suggest that a bias has likely occurred in the former, as patients in better conditions (and consequently better prognosis) are suitable to primary tumor resection.

### Limitations

Selection bias and potential confounders were limitations of this study. Because there are not enough clinical data, we did not analyze the subgroup on the left and right colorectal cancer, staging, differentiation degree or whether only liver metastasis. Moreover, more RCT studies are needed to further verify this conclusion.

## Data Availability Statement

The original contributions presented in the study are included in the article/supplementary material. Further inquiries can be directed to the corresponding authors.

## Author Contributions

Data extraction was conducted independently by YS and LX, and discrepancies were resolved by WY and XX before the final analysis. SZ revised the work critically for important intellectual content.

## Conflict of Interest

The authors declare no potential conflict of interest.

## Publisher’s Note

All claims expressed in this article are solely those of the authors and do not necessarily represent those of their affiliated organizations, or those of the publisher, the editors and the reviewers. Any product that may be evaluated in this article, or claim that may be made by its manufacturer, is not guaranteed or endorsed by the publisher.
